# Biomarkers for Alcohol Use and Abuse: A Summary

**Published:** 2004

**Authors:** Karen Peterson

**Affiliations:** Karen Peterson, Ph.D., is chief of the Research Policy and Special Programs Branch in the National Institute on Alcohol Abuse and Alcoholism’s Office of Scientific Affairs, Bethesda, Maryland

**Keywords:** screening and diagnostic method for AOD (alcohol and other drug) use, pattern of AOD use, alcohol-related biological markers, alcohol-related biochemical markers, alcohol-related genetic markers

## Abstract

Clinicians can use several biochemical measurements to objectively assess patients’ current or past alcohol use. However, none of these currently available biomarkers—including measures of various liver enzymes and blood volume—are ideal. Several more experimental markers hold promise for measuring acute alcohol consumption and relapse. These include certain alcohol byproducts, such as acetaldehyde, ethyl glucuronide (EtG), and fatty acid ethyl esters (FAEE), as well as two measures of sialic acid, a carbohydrate that appears to be altered in alcoholics. Some progress has been made in finding markers that predict people’s genetic predisposition to alcoholism, such as genetic differences in several neurotransmitters, including beta-endorphin and gamma-aminobutryic acid (GABA).

To treat people with alcoholism adequately, clinicians need tools that can properly assess not only the extent of the patients’ recent and past drinking activity but also any family history of drinking problems (i.e., genetic predispositions to alcohol abuse and alcoholism) they may have. A good case history is certainly a start, but more objective measures also are important. Biochemical substances in the body that can indicate the presence or progress of a condition, or any genetic predisposition toward it, are called biomarkers. There are two kinds: state markers and trait markers. State markers are biochemical measures that tell clinicians something about people’s recent drinking patterns, including whether they have a history of heavy drinking and whether they have had a recent binge or even just a few drinks. Trait markers are biochemical markers that reveal something about a person’s inherited risk of abusing alcohol. A good biomarker, whether state or trait, should be sensitive—that is, accurate for most if not all drinkers, not just a subset—and specific, or linked to alcohol use but not other illnesses or problems. The test used to measure the biomarker also should be precise and accurate. (For definitions of terms central to this article, see the accompanying Glossary.)

This article presents an overview of current alcohol biomarkers. Although some of the markers described here have been in wide use for many years, some are new and under development. The goal is to compile a large toolkit of biomarkers that can provide objective, quantitative data to clinicians as they evaluate patients.

## State Markers

When clinicians evaluate a patient’s history of alcohol consumption, they want to know not only about recent (i.e., acute) drinking patterns but also about long-term (i.e., chronic) drinking patterns and whether that drinking has been moderate or heavy. (Heavy drinking is defined in this article as consuming more than 60 grams of alcohol—between four and five drinks—a day, unless otherwise noted. Consuming this amount for 2 weeks or more is considered to be chronic heavy drinking.)

The various state biomarkers use several techniques to assess different levels or periods of alcohol consumption. These markers may be related to chemicals produced when the body breaks down, or metabolizes, alcohol or may reflect changes in other compounds, cells, or tissues that result from chronic or acute alcohol exposure. (The table summarizes the sensitivity, specificity, and potential uses of these measures. For further information on state markers, see the figure.)

### Currently Used State Markers

For many years, clinicians have had access to a group of biomarkers that indicate a person’s alcohol intake. Several of these reflect the activity of certain liver enzymes: serum gamma-glutamyltransferase (GGT), aspartate aminotransferase (AST), alanine aminotransferase (ALT), and carbohydrate-deficient transferrin (CDT), a protein that has received much attention in recent years. Another marker, n-acetyl-β-hexosaminidase (beta-Hex), indicates that liver cells, as well as other cells, have been breaking down carbohydrates, which are found in great numbers in alcohol (Javors and Johnson 2003). Clinicians also have used red blood cell volume, known as mean corpuscular volume (MCV), as a biomarker of alcohol intake.

#### Gamma-Glutamyltransferase (GGT)

This glycoprotein—a large molecule made up of both proteins and carbohydrates—aids in digestion and is found in key liver cells (or hepatocytes) and in other cells involved in bile production, including biliary epithelial cells. Elevated GGT levels are an early indicator of liver disease; chronic heavy drinkers, especially those who also take certain other drugs, often have increased GGT levels. However, GGT is not a very sensitive marker, showing up in only 30–50 percent of excessive drinkers in the general population (Conigrave et al. 2003). Nor is it a specific marker of chronic heavy alcohol use, because other digestive diseases, such as pancreatitis and prostate disease, also can raise GGT levels.

***Aspartate Aminotransferace (AST) and Alanine Aminotransferace (ALT)*** are enzymes that help metabolize amino acids, the building blocks of proteins. They are an even less sensitive measure of alcoholism than GGT; indeed, they are more useful as an indication of liver disease than as a direct link to alcohol consumption. Nevertheless, research finds that when otherwise healthy people drink large amounts of alcohol, AST and ALT levels in the blood increase (Halvorson et al. 1993). Of the two enzymes, ALT is the more specific measure of alcohol-induced liver injury because it is found predominantly in the liver, whereas AST is found in several organs, including the liver, heart, muscle, kidney, and brain. Very high levels of these enzymes (e.g., 500 units per liter) may indicate alcoholic liver disease. Clinicians often use a patient’s ratio of AST to ALT to confirm an impression of heavy alcohol consumption. However, because these markers are not as accurate in patients who are under age 30 or over age 70, they are less useful than some of the other more comprehensive markers (Halvorson et al. 1993).

***Mean Corpuscular Volume (MCV)***, a person’s volume of red blood cells, also is associated with heavy chronic drinking (Neumann and Spies 2003), as the MCV in heavy drinkers tends to exceed the average range. This marker is less useful clinically, however, because the MCV stays high for several months after a person stops drinking, so someone could be abstinent but still show a high MCV value. In addition, other conditions may affect MCV, reducing its specificity and further confounding any interpretation of results using this marker.

**n*-Acetyl-β-Hexosaminidase (Beta-Hex)***, an enzyme found to be elevated in heavy drinkers (Javors and Johnson 2003), has been shown in some early studies to be both a sensitive and specific measure of heavy drinking. In addition, unlike MCV, the increased beta-Hex subsides to normal levels after only 7 to 10 days of abstinence. However, the beta-Hex assay is difficult to obtain in the United States, so clinicians have little experience using it with different treatment populations. Other conditions, such as diabetes and hypertension, also appear to elevate beta-Hex.

***Carbohydrate-Deficient Transferrin (CDT)*** is a version of the glycoprotein transferrin, a molecule responsible for carrying iron within the bloodstream. CDT, as its name implies, is a form of this molecule that is deficient in the carbohydrate sialic acid. Normally, transferrin contains four to six sialic acid molecules, but research indicates that drinking disrupts sialic acid’s ability to attach to transferrin as well as other molecules. Many versions of transferrin normally are found in healthy people, but studies indicate that heavy drinkers have higher amounts of the CDT version than nondrinkers.

**Glossary**
Biomarker: A biochemical feature (a compound or series of compounds) that can be used to measure the progress of a disease or the effects of treatment.
State marker: A *biomarker* that provides information about recent drinking activity.
Trait marker: A *biomarker* that provides information about a person’s genetic predisposition toward alcohol dependence.
Accuracy: A measure’s ability to obtain the expected result.
Precision: A measure’s “repeatability,” or ability to repeat the expected result without significant day-to-day variation.
Sensitivity: A test’s ability to detect small differences in concentration of the *biomarker*.
Specificity: A test’s ability to indicate the absence of a *biomarker* in a sample that is truly negative for that *biomarker*.

CDT has been widely used by clinicians in recent years to screen for heavy alcohol consumption. Although it appears to be a highly specific measure of alcohol consumption, showing low rates of false positives, CDT is difficult to measure accurately. Distinguishing CDT from other forms of transferrin is challenging. In addition, even teetotalers have low concentrations of CDT in their blood, and people with generally high concentrations of total transferrin will necessarily have high absolute numbers of CDT molecules, regardless of their drinking status. These difficulties affect the precision and therefore the sensitivity of the test. Another disadvantage with the CDT marker is that there is a relatively high rate of false negative results: Some patients who drink heavily do not show elevated levels of CDT. Researchers also find that, in general, women tend to have higher CDT levels than men, regardless of their drinking history (Arndt 2000). What causes this gender difference is not clear, but some tests now on the market claim to correct for it.

Recently, researchers have made several improvements in measuring CDT, including developing agents that specifically detect CDT (i.e., immunological reagents) (Bean et al. 2001) and measuring CDT levels as a percentage of total transferrin, rather than an absolute value (Anttila et al. 2004). Some studies also have examined the usefulness of combining the CDT and GGT tests (Chen et al. 2003), finding that, at least in men, using both tests is more sensitive than using either marker alone.

Despite the disadvantages of the CDT marker, it remains a very well-characterized biomarker for heavy alcohol intake.

### Newer State Markers Under Study

Several new markers for assessing alcohol intake and alcohol abuse are at various stages of research and development, including the plasma sialic acid index of apolipoprotein J (SIJ), total serum sialic acid (TSA), 5-hydroxytryptophol (5-HTOL), and various fatty acid ethyl esters (FAEEs). None of these tests are commercially available, but some look promising, as described in the following section.

**Figure:**
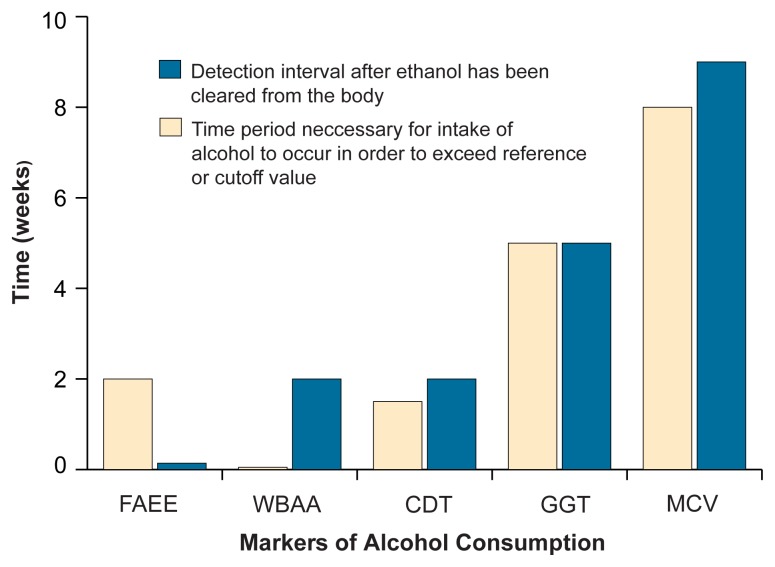
A comparison of some state markers of alcohol consumption. Bars represent approximations, and some variability exists for each marker time period because of individual variability, different test manufacturers, and the like. FAEE = fatty acid ethyl esters, WBAA = whole blood–associated acetaldehyde, CDT = carbohydrate-deficient transferrin, GGT = gamma-glutamyltransferase, MCV = mean corpuscular volume.

#### Plasma Sialic Acid Index of Apolipoprotein J (SIJ)

Apolipoprotein J is a glycoprotein found in needed complexes (i.e., lipoproteins) that are responsible for transporting fats (i.e., lipids) in the blood. Research indicates that apolipoprotein J may help transfer fats such as cholesterol from one lipoprotein to another (Javors and Johnson 2003). Like the molecule transferrin, apolipoprotein J contains sialic acid particles that may be reduced in number after alcohol consumption. Apolipoprotein J has more than four times more sialic acid chains than transferrin, making it easier to measure changes in sialic acid content caused by heavy alcohol consumption. More study is needed, but preliminary findings show promise for SIJ as a highly specific and easy-to-measure marker.

#### Total Serum Sialic Acid (TSA)

Because of sialic acid’s clear potential as a highly specific marker for alcohol use, researchers have begun to study the potential of measuring total sialic acid (TSA) levels in patients’ blood, rather than looking at the difference in sialic acid chains only on glycoproteins such as transferrin and apolipoprotein J. Early studies (Javors and Johnson 2003) demonstrate that, compared with social drinkers of both genders, both male and female alcoholics had elevated amounts of TSA. The test for TSA has similar sensitivity and specificity to the test for CDT for measuring alcohol consumption. However, because TSA levels take longer than either CDT or GGT to decrease during periods of abstinence, the TSA test might not be as useful for treatment programs assessing patients for relapse.

#### 5-Hydroxytryptophol (5-HTOL)

When the body breaks down a key chemical involved in cellular communication in the brain (the neurotransmitter serotonin), one of the minor resulting components (or metabolites) is 5-hydroxytryptophol. Alcohol and its primary breakdown product, acetaldehyde, affect the metabolism of serotonin so that the body produces more 5-HTOL when people consume alcohol than when they do not drink. The body disposes of 5-HTOL via the urine, where it can be detected for approximately 5 to 15 hours longer than standard alcohol measurements, which can detect alcohol in the urine for a little over an hour for each drink consumed (Beck and Helander 2003). Because of its ability to detect people’s alcohol use for up to 24 hours after they have been drinking, 5-HTOL is considered a 24-hour biomarker for heavy alcohol consumption. Although the marker requires more study, preliminary work indicates that it is both sensitive and specific for detecting recent heavy alcohol consumption (Beck and Helander 2003). Testing for 5-HTOL may prove especially useful in forensic toxicology. Emergency room clinicians may find it helps detect people who consumed large amounts of alcohol before preparation for surgery, and treatment professionals may be able to use this test to monitor the care of people involved in treatment maintenance programs (although the antidrinking medication disulfiram, which people in these settings may be taking, also can lead to increases 5-HTOL levels). In addition, research has shown that the ratio of 5-HTOL to another serotonin metabolite, 5-HIAA or 5-hydroxyindole-3-acetic acid, is a useful indication of previous drinking (Johnson et al. 2004).

**Table:** Summary of State Markers for Alcohol Consumption

Marker	Sensitivity (percent)	Diagnostic Specificity (percent)	Possible or Current Use	Used Clinically in U.S.?
Gamma-glutamyltransferase (GGT)	61^1^	n/a	Chronic alcohol abuse	Yes
Alanine aminotransferase (ALT)	Method-dependent	n/a	Chronic alcohol abuse	Yes
Aspartate aminotransferase (AST)	56^1^	n/a	Chronic alcohol abuse	Yes
Carbohydrate-deficient transferrin (CDT)	26–83^2^*	92^3^	Heavy alcohol use**	Yes
N-acetyl-β-hexosaminidase	94^2^	91^2^	Heavy alcohol use	No
Whole blood–associated acetaldehyde (WBAA)	100^4^	95^4^	Recent alcohol consumption at all levels; monitoring abstinence	Yes
Mean corpuscular volume (MCV)	47^1^	n/a	Heavy alcohol use	Yes
Apolipoprotein J	n/a	n/a	Heavy alcohol use	No
5-hydroxytryptophol (5-HTOL)	n/a	n/a	Monitoring sobriety	No
Salsolinol	n/a	n/a	Chronic alcohol consumption	No
Fatty acid ethyl esters (FAEE)	100^5^	90^5^	Recent heavy alcohol use	No
Ethyl glucuronide (EtG)	n/a	Method-dependent	Monitoring sobriety; forensics	No

* Depending on method and gender

** More than 60 grams per day (4–5 standard drinks) n/a = data not available

1 Anttila et al. 2004.

2 Stowell et al. 1997.

3 Javors and Johnson 2003.

4 Bean et al. 2001.

5 Wurst et al. 2004.

#### Fatty Acid Ethyl Esters (FAEE)

Along with acetaldehyde, the body also produces FAEE when it breaks down alcohol. FAEE is measured as a combination of four separate molecules and is found in the liver, pancreas, and fat (i.e., adipose) tissues up to 24 hours after alcohol consumption. FAEE is a sensitive and specific marker for distinguishing social drinkers from heavy or alcohol-dependent drinkers (Wurst et al. 2004; Salem et al. 2001). Because it also is found in human hair (Wurst et al. 2003; Yegles et al. 2004), some researchers suggest using FAEE in hair as a marker for chronic heavy alcohol consumption (Wurst et al. 2004). The body cannot flush FAEE out of hair, so the compound builds up over a long period of chronic drinking.

FAEE measured in liver and adipose tissue also has been used as a postmortem marker of alcohol consumption (Salem et al. 2001). Such a measure is needed because current measures, such as blood alcohol levels, can be artificially high as a result of alcohol formation in the body after death. So far, FAEE looks promising. Preliminary studies (Salem et al. 2001) show that when measured in adipose tissue, FAEE is useful as a biomarker up to 12 hours after death in alcohol-treated animals; when measured in animal liver tissue, FAEE is useful up to 24 hours after alcohol treatment. Further study is required to fully explore FAEE’s sensitivity and specificity.

#### Ethyl Glucuronide (EtG)

EtG is another minor metabolite of alcohol that forms in the liver when alcohol reacts with glucuronic acid, a substance which works to detoxify drugs by turning them into water-soluble compounds that can be easily removed from the body.

EtG can be detected in the blood for up to 36 hours and in the urine for up to 5 days after heavy alcohol use. In addition to blood and urine, EtG is detectable in other body fluids, hair, and body tissues (Wurst et al. 2003), although no apparent correlation has been found between alcohol consumption and the presence of EtG in hair. When people test positive for EtG, it is likely that they consumed alcohol recently, even if there is no alcohol left in their bodies. This makes EtG especially useful for detecting drinking relapses. Measuring EtG levels is difficult, however. A rather sophisticated instrument, the mass spectrometer, is required for an accurate reading of EtG from urine. And so far, attempts to produce a measure for urine-based EtG using simpler techniques or to measure EtG in other body fluids or hair have yielded less than satisfactory results (Wurst et al. 2003). Once an easy method of measuring EtG is developed, it has potential to detect relapse, to detect alcohol use in settings where drinking is deemed risky, such as for testing those who drive vehicles, fly planes, or operate other sophisticated machinery, as well as for forensic use.

#### Acetaldehyde

The first compound the body produces as it metabolizes alcohol is acetaldehyde, which exists on its own and also can bind to certain proteins, including hemoglobin (a protein in red blood cells that carries oxygen). Researchers are able to measure concentrations of both free and bound acetaldehyde in blood samples using high-performance liquid chromatography and fluorescence detection—known as the whole blood–associated acetaldehyde assay (WBAA) (Halvorson et al. 1993). This assay is highly specific, extremely sensitive (Peterson and Polizzi 1987), and has excellent precision. The insurance testing industry has used WBAA for more than a decade to test for heavy alcohol consumption (FDA approval for wider clinical use is pending). Its potential is even greater as a clinical tool to monitor people in alcoholism treatment programs, because this test can provide a picture of alcohol use over time. This works because, as a person continues to drink, hemoglobin-bound acetaldehyde accumulates in red blood cells over their 120-day average life span, and this buildup shows up as an increasing WBAA assay number. Levels of protein-bound acetaldehyde remain high for approximately a month after alcohol consumption (Halvorson et al. 1993). The ability of the WBAA assay to measure alcohol consumption patterns over time makes it unique among the biomarkers described here.

#### Salsolinol

This compound, formed when the neurotransmitter dopamine reacts either with alcohol’s byproduct acetaldehyde or with pyruvate (a metabolite of glucose that is used by cells for energy), shows some promise as a state marker for chronic alcohol consumption. However, the usefulness of salsolinol may depend on how it is measured—whether, for example, in blood, urine, or brain tissue. Salsolinol levels in urine have been found to decrease following *acute* alcohol consumption (Haber et al. 2002), and measuring salsolinol levels in the blood may provide a better indication of *chronic* alcohol consumption. A study by Haber and coworkers (Haber et al. 2002) showed that, compared with nonalcoholics, alcoholics who have been abstinent for as little as 1 week have decreased salsolinol levels in one type of white blood cell (lymphocytes). Studies of salsolinol levels in the brain, in contrast, found no difference in salsolinol levels between alcoholics and nonalcoholics (Musshoff et al. 2005). This may indicate problems of measuring salsolinol in the brain as well as inherent differences in salsolinol levels among different biological sources.

### Proteomics

Researchers have begun to use proteomics, the systematic study of proteins that are matched to certain known genes, to search for biomarkers of alcohol consumption. Recently, investigators used a powerful technique, surface-enhanced laser desorption/ionization–time of flight–mass spectrometry (SELDI-TOF-MS), to study serum samples from alcoholics who had consumed more than 10 drinks a day for at least 10 years (Nomura et al. 2004). The researchers examined the protein profile in the blood of these people upon admission to an alcoholism treatment program and again after abstinence—taking measures throughout the treatment program. They found measurable differences in the levels of two proteins, a fragment of the fibrinogen αE chain and a fragment of apoprotein A–II. Specifically, patients had low levels of the proteins when they were drinking and significantly increased levels starting as soon as 1 week into the treatment program. Nomura and colleagues (2004) concluded that the two protein fragments have potential as markers of excessive alcohol consumption in heavy drinkers seeking treatment.

## Trait Markers

Biochemical markers are being developed to identify people with a genetic predisposition to alcohol abuse and alcoholism. Knowing who is at risk can help prevent alcohol problems altogether, or enable a person to seek early treatment for developing problems or to experience better treatment outcomes. At a minimum, any useful trait marker should satisfy at least three criteria: It should be passed down from parents to children through the genes (i.e., be heritable), it should be associated with the disease in question in the general population, and it should be independent of the status of the disease, meaning that it would be present whether the person displayed symptoms of the disease or was asymptomatic (Ratsma et al. 2002). Biomarkers that meet these criteria and show low rates of false positive and false negative results will have excellent value in predicting the likelihood that a person will develop alcohol dependence. Much of the research in this area is preliminary, but several markers, including an enzyme and a group of neurotransmitters, hint at its potential.

### Adenylyl Cyclase (AC) Activity

A protein found in cell membranes, AC plays an important role in providing the cell with energy. Researchers became interested in AC activity as a potential trait marker when they discovered that AC activity is inherited and the enzyme is less active in the blood platelet cells of abstinent alcoholics than in nonalcoholics (Hoffman et al. 2002). In addition, AC activity increases when alcoholics begin drinking again, suggesting that alcohol somehow stimulates AC activity. Unfortunately, it appears that marijuana and other drug use also affect AC activity, making it an imprecise marker for alcohol use specifically (Hoffman et al. 2002). Researchers now are searching for possible differences between alcoholics and nonalcoholics in the structure of genes associated with AC activity.

### Gamma-Aminobutyric acid (GABA)

The neurotransmitter GABA is a chemical that acts on special docking molecules (i.e., receptors) in brain cells (i.e., neurons) for the GABA molecule. These molecules enable charged chlorine ions to enter and exit the cell, thus controlling the chemical balance of the cells. Studies (Ratsma et al. 2002; Mihic and Harris 1996) find that people have different levels of GABA and these differences are inherited. In addition, studies show that people who are alcohol dependent have lower levels of GABA than do non-alcohol-dependent people. Thus, at least in these preliminary studies, GABA fulfills two of the three requirements of a trait marker for alcoholism.

### Dopamine

Another neurotransmitter, dopamine, acts at the receptor level and is believed to be involved in the brain’s reward system. A recent study (Ratsma et al. 2002) found that male alcoholics who had been abstinent for 7 years showed a lower level of dopamine receptor activity compared with nonalcoholic men, whereas a previous study (Balldin et al. 1985) demonstrated that alcoholics, after a withdrawal period of 4 to 7 days, showed an elevated response to dopamine, indicating elevated receptor activity. Other studies examining levels of the major byproduct of dopamine metabolism, homovanillic acid, also have had contradictory results. Some investigators (George et al. 1999) found higher levels of homovanillic acid in alcoholics compared with nonalcoholics, but other researchers (Petrakis et al. 1999) found lower levels for alcoholics. Because of these conflicting baseline findings, dopamine is not considered a good candidate trait marker at this time.

### Beta-Endorphin

The neurotransmitter beta-endorphin is an opioid produced by the pituitary gland. It works to activate neurons’ opiate receptors and is thought to produce natural pain relief and a feeling of exhilaration. Studies find that alcoholics have lower levels of beta-endorphin than nonalcoholics, and that children of alcoholics have fewer opioid receptors than children of nonalcoholics (Volpicelli et al. 1990; Zalewska-Kaszubska and Czarnecka 2005; Oswald and Wand 2004). These findings indicate that differences in beta-endorphin levels are both specific to alcoholism and inherited, fulfilling two of the three requirements for a trait marker of alcoholism. Researchers still need to do much more work to establish beta-endorphin as a true trait marker.

### Serotonin

Preliminary research indicates that the neurotransmitter serotonin or other biochemicals associated with serotonin show potential as trait markers for alcoholism. One such biochemical—the amino acid tryptophan, which influences how much serotonin the brain produces—may be decreased in people consuming excess alcohol (Ratsma et al. 2002; Swann et al. 1999).

Another line of research examines the activity of the serotonin transporter, which controls how much serotonin is available to cells. Research finds natural differences among people in serotonin transporter activity in blood platelets, and these differences appear to be inherited. In addition, alcoholics who have been abstinent for extended periods of time show higher serotonin transporter activity than nonalcoholics, as do children of alcoholics compared with children of nonalcoholics (Oroszi and Goldman 2004). These findings indicate that serotonin transporter activity in blood platelets has potential as a trait marker for alcoholism.

## Summary

The search for ideal biomarkers of alcohol consumption (state) and for the genetic predisposition toward alcohol dependence (trait) continues. Although the state markers currently in use have value, their limitations and weaknesses make it desirable to develop more sensitive and specific markers. The importance of a marker’s precision, accuracy, sensitivity, and specificity cannot be overstated. Although it is likely that researchers never will find one marker to satisfy all clinical needs, they eventually may develop combinations of markers for different clinical purposes, from unselective screening (i.e., drinking versus not drinking) to confirming a suspicion of alcohol abuse or dependence. In addition, proteomics may provide “signatures,” or combinations of compounds that are important diagnostically.

Alcohol consumption patterns, like most human behavior, are complex. Clinicians often need to detect patterns of drinking other than the chronic, heavy drinking detected by GGT, AST, ALT, and CDT. For example, they may need to know whether a person has done any amount of drinking recently or what type of drinking has occurred (e.g., heavy or social drinking). Therefore, finding new biomarkers that measure many different aspects of alcohol consumption will vastly increase the clinician’s ability to detect and treat alcohol abuse and dependence. In addition, such biomarkers will help provide more precise definitions of alcohol consumption and alcohol use disorders not only in the clinic but in research where these terms currently are defined less precisely, such as number of drinks consumed over a certain period of time.

Finally, more research is necessary before clinically useful trait markers of genetic predisposition to alcohol dependence are fully developed. The markers first must be validated clinically by testing people before they develop alcoholism and waiting to see how well the marker predicts later behavior. As researchers further develop the markers described here and discover more biomarkers, their work should greatly improve clinicians’ ability to objectively assess alcohol consumption as well as genetic predisposition to alcohol use disorders.
